# The soil crisis: the need to treat as a global health problem and the pivotal role of microbes in prophylaxis and therapy

**DOI:** 10.1111/1751-7915.13771

**Published:** 2021-03-10

**Authors:** Kenneth Timmis, Juan Luis Ramos

**Affiliations:** ^1^ Institute of Microbiology Technical University Braunschweig Braunschweig Germany; ^2^ Estacion Experimental de Zaidin CSIC Granada Spain

## Abstract

Soil provides a multitude of services that are essential to a healthily functioning biosphere and continuity of the human race, such as feeding the growing human population and the sequestration of carbon needed to counteract global warming. Healthy soil availability is the limiting parameter in the provision of a number of these services. As a result of anthropogenic abuses, and natural and global warming‐promoted extreme weather events, Planet Earth is currently experiencing an unprecedented crisis of soil deterioration, desertification and erosive loss that increasingly prejudices the services it provides. Such services are pivotal to the Sustainability Development Goals formulated by the United Nations. Immediate and coordinated action on a global scale is urgently required to slow and ultimately reverse the loss of healthy soils.

Despite the ‘dirt‐dust’, non‐vital appearance of soil, it is a highly dynamic living entity, whose life is overwhelmingly microbial. The soil microbiota, which constitutes the greatest reservoir and donor of microbial diversity on Earth, acts as a vast bioreactor, mediating a myriad of chemical reactions that turn the biogeochemical cycles, recycle wastes, purify water, and underpin the multitude of other services soil provides. Fuelling the belowground microbial bioreactor is the aboveground plant and photosynthetic surface microbial life which captures solar energy, fixes inorganic CO_2_ to organic carbon, and channels fixed carbon and energy into soil.

In order to muster an effective response to the crisis, to avoid further deterioration, and to restore unhealthy soils, we need a new and coherent approach, namely to deal with soils worldwide as patients in need of health care and create (i) a public health system for development of effective policies for land use, conservation, restoration, recommendations of prophylactic measures, monitoring and identification of problems (epidemiology), organizing crisis responses, etc., and (ii) a healthcare system charged with soil care: the promotion of good practices, implementation of prophylaxis measures, and institution of therapies for treatment of unhealthy soils and restoration of drylands. These systems need to be national but there is also a desperate need for international coordination. To enable development of effective, evidence‐based strategies that will underpin the efforts of soil healthcare systems, a substantial investment in wide‐ranging interdisciplinary research on soil health and disease is mandatory. This must lead to a level of understanding of the soil:biota functionalities underlying key ecosystem services that enables formulation of effective diagnosis‐prophylaxis‐therapy pathways for sustainable use, protection and restoration of different types of soil resources in different climatic zones. These conservation‐regenerative‐restorative measures need to be complemented by an educative‐political‐economic‐legislative framework that provides incentives encouraging soil care: knowledge, policy, economic and others, and laws which promote international adherence to the principles of restorative soil management. And: we must all be engaged in improving soil health; everyone has a duty of care (https://www.bbc.co.uk/ideas/videos/why‐soil‐is‐one‐of‐the‐most‐amazing‐things‐on‐eart/p090cf64). Creative application of microbes, microbiomes and microbial biotechnology will be central to the successful operation of the healthcare systems.

## Soil = dirt: it has an image problem (‘*For all things come from earth, and all things end by becoming earth’*. Xenophanes of Colophon, b ca 580 BC)

Soil, earth, dirt and mud are something we get on our clothes when we fall, and send it off with little ceremony and no thought to the washing machine to be removed and discarded into the waste water system. This is similar to our discarding mouldy bread or smelly fish into the trashcan, where it disappears from sight and is duly transported to wherever food ends up that has first been eaten by spoilage microbes. Soil has an image problem, as do microbes. A few pathogens have given microbes the disparaging name *germs*; the mud on our jeans, the soil under our fingernails, has given soil the disparaging name *dirt*. But all terrestrial life forms have their origins, lifespans and end‐of‐the‐road recycling in soil; our relationship with soil is much more intimate than we may think. And soil is precious, and becoming more precious by the day, as we abuse and lose it, and place ever‐increasing, sometimes overwhelming, demands on it. It is precious because, on one hand, it provides us and rest of the biosphere with a myriad of vital goods and services, and, on the other, it is a rate‐limiting resource for the provision of just those goods and services, a resource that is inexorably reducing over time. Crucially, dirt and germs are physically and functionally intimately intertwined and interdependent: soil would be essentially lifeless – unable to provide its essential key services – without germs, and most germs would not have a home without dirt: they are obligate partners steering and mediating so many pivotal biosphere activities upon which we depend.

*Soil is alive and a key enabler of life on land and in the air. The images of soil and of microbes need to change to reflect the benefits they bestow upon us and the rest of the biosphere, to promote humankind to embrace the policies and actions needed to repair, restore and maintain in good health soils that are currently unhealthy, and to attain soil sustainability*.

## Soil is the thin, fragile, non‐renewable skin of the planet and home to the terrestrial biosphere. (‘*A cloak of loose, soft material, held to the earth’s hard surface by gravity, is all that lies between life and lifelessness’*. Fuller, 1975)

Soil is the thin, threadbare underwear beneath the outer phytogarments that decorate the rocky land surface of planet Earth (https://solarsystem.nasa.gov/planets/in‐depth/). It consists of a mineral matrix, formed by the weathering of rocks by diverse physical, chemical and biological activities, an amazing range of organisms living in or on it, and all the organic materials they deposit. Algae and cyanobacteria on the soil surface, and diverse belowground microbes, especially the well‐fed ones living around plant roots, produce polymers that glue soil particles together, create soil crumbs and stabilize the soil matrix. It also contains stones and rocks, so is physically heterogeneous. Soil thicknesses range from microns on seemingly bare rock to tens of metres, though typically are less than three metres thick (Richter and Markewitz, 1995). When this depth is contrasted with the > 6000 km radius of the planet, it becomes apparent just how thin the layer of soil, and the life it supports, is. Although rock weathering and soil formation are continuous processes, they are slow: generation of 3 mm of topsoil takes a century, so topsoil is considered to be a non‐renewable resource.

## Soil is dynamic

The abiotic component of the soil is of itself (i.e. independently of external forces like wind, rain and floods, and engineering and agricultural activities of humankind), rather static. However, the enormous diversity of life that soil houses endows it with considerable dynamism. Soil serves as the substratum for plants, providing them with anchorage, and the water and minerals needed for phototrophic growth. Plant roots, which make up one third of all planetary phytobiomass (Robinson, 2007), grow through soil, actively exploring it for nutrient resources (e.g. Cahill and McNickle, 2011; Cabal *et al*., 2020), and tree roots can exert great strength in moving soil around (witness paths and roads lifted by tree roots). Some animals, like earthworms, are designated soil engineers because they have such a major impact on soil by continuously moving through it, thereby creating the drillosphere. In so doing, they mix and aerate soil, taking it into their digestive tracts and, together with their gut microbes, contribute to nutrient cycling, thereby enhancing a number of soil properties, including its porosity and microbial diversity (Nechitaylo *et al*., 2010; Blouin *et al*., 2013). Termites are another example of ecosystem engineers. Less comprehensive soil modifiers are animals like tunnelling moles, that chase soil invertebrates for food, and other animals that create lairs in soil, like rodents, rabbits, foxes and so on. Then, there are animals that ordinarily have a lower impact, such as many invertebrates and birds that spend only part of their lives on the surface.

## Soil is a major planetary resource providing goods and services crucial to global sustainability

Soil provides a multitude of services (https://doi.org/10.4060/cb1928en; https://millenniumassessment.org/documents/document.300.aspx.pdf; Bünemann *et al*., 2018) essential to planetary and human sustainability, as articulated by the United Nations in their Sustainability Development Goals (SDGs; https://sustainabledevelopment.un.org/post2015/transformingourworld; https://doi.org/10.4060/cb1928en). Just to list a few examples:

1. *Substratum and nutrient provider of plants*. It provides anchorage and nourishment for plants (*SDGs 2, 7*). Plants provide primary food to us and our food animals, so we can eat and grow, wood so we can construct homes (and, before the era of global warming, heat them)/create pianos/violins/guitars/paper for books and paintings/cricket–baseball bats and goalposts/fencing/etc., thatch for roofing/insulation to keep us warm and dry, with lawns and pitches, so we can play soccer, rugby, cricket, tennis, with exquisitely beautiful and varied flowers for vegetation artistry, so that we can create and enjoy lovely gardens and flower arrangements in our homes/places of worship/institutions, vegetable dyes like indigo to colour our clothes and bodies, and so on. Firewood used for heating and cooking has in the meantime been replaced by plants grown to create biofuel renewable forms of energy.
2. *Essence of agriculture* (‘*To be a successful farmer one must first know the nature of the soil’*. Xenophon, The Oeconomicus, ~ 400 B.C.*)*. Most of the food we eat is produced by farming, most of it through the cultivation in soil of domesticated plant crops which are either used directly as food, or used to feed domesticated animals that become meat (though fish production by aquaculture is increasing in importance). As the human population grows – the population is estimated to reach 10 billion by 2050 – the pressure builds to produce ever‐increasing amounts of food, as expressed in *SDG 2*: *End hunger*.
  But increased food production must not be achieved through unsustainable exploitation of natural resources that provide ecosystem services vital for human and biosphere survival. Feeding the human population requires healthy soils and sustainable agricultural practices aligned with strategies to mitigate climate change.
*Soil is a vital resource for agriculture and the feeding of the world population. In the absence of aid, insufficient local food production in low‐income countries can create famine and is a catalyst for mass human migrations*.
3. *Largest terrestrial reservoir of water*. Soil is porous and under normal levels of water saturation and humidity, its pores hold vast amounts of water. Globally, soil is the largest reservoir of freshwater, holding two thirds of all freshwater on the planet (https://www.isric.org/utilise/global‐issues/water). This porewater supplies aboveground plants with the water they need for growth, and that they extract through their belowground roots (*SDG 2*), and the water needs of other soil‐dwelling organisms.
4. *Flood and landslide regulator*. Most soils have residual water‐holding capacity, and can accommodate significant amounts of new precipitation and run‐off, thereby counteracting flooding tendencies, particularly on flood plains. On the other hand, much land consists of slopes on which soil and rock cover are susceptible to landslides. The water absorptive capacity of soil can result in substantial gains in soil weight per volume during heavy rainfall that, in combination with the action of water run‐off, increase the tendency to initiate landslides. However, the root systems of tree cover of slopes stabilize soils and counteract landslide initiation.
5. *Provider of materials*. Soil provides materials. Clay‐rich soil is used to produce bricks for construction (*SDG 11*), and clay itself is used to produce pottery/earthenware/porcelain utility and decorative/artistic items.
6. *Provider of biological surfaces*. Soil provides the surface for many recreational activities that contribute to physical and mental well‐being (*SDG 3*). Much of the recreation time we spend out of doors is in parks, school/university/community sports fields, country paths, hiking and horse‐riding trails, most of which are covered in grass.
7. *Enabler of enterprise and employment*. Soil is an important employer (*SDGs 1, 8*). Prior to the industrial revolution, farming was the main occupation of people in most societies. Since then, mechanization has reduced the proportion of people working on the land but, globally, agriculture and food animal husbandry is still a very important source of employment (e.g. https://www.statista.com/statistics/271320/distribution‐of‐the‐workforce‐across‐economic‐sectors‐in‐india/; https://data.worldbank.org/indicator/SL.AGR.EMPL.ZS). Moreover, soil‐ and soil:plant‐related occupations, such as maintenance of public and recreational areas, gardening, waste treatment by composting/wetlands constructed for wastewater treatment (e.g. see Vimazal, 2011), etc., provide significant employment.
8. *Home to a vast array of wildlife*. Soil houses an incredible spectrum of largely unseen and hence unappreciated biodiversity (http://www.fao.org/documents/card/en/c/CB1928EN/), and of important food webs that maintain such diversity (*SDG 15*), and keep the biogeochemical cycles turning (Crowther *et al*., 2019). Microbes are important functional constituents of this diversity (FAO, 2020). For example, nitrogen‐fixing soil microbes, either symbiotic or free‐living, provide plants with growth‐limiting nitrogen in a usable form, rhizosphere microbes render insoluble and unavailable plant nutrients, like phosphorus and some trace elements, soluble and available. Others produce secondary metabolites that inhibit root pathogens or hormones that stimulate plant growth (e.g. see Roca *et al*., 2013; *SDG 2* **)**.
9. *Driver of microbial diversification, home to a vast range of microbial life and the most important reservoir of microbial diversity*. Soil is also home to an incredible spectrum of microorganisms: bacteria, archaea, fungi, protists, algae and their viruses (*SDG 15*). Soil is particulate, granular, discontinuous and patchy, with a myriad of spatially distinct micro‐environments characterized by different physico‐chemical conditions existing side‐by‐side that provide a multitude of discrete and different microbial habitats (e.g. see O’Brian *et al*., 2016; Fierer, 2017). These physico‐chemical microhabitat differences may themselves become amplified by the life in the soil, because different microbial communities flourish under different conditions, and modify their environments in different ways that favour their own growth and disfavour the growth of others, thereby amplifying soil heterogeneity.
  The biosphere has been described as habitats consisting of gradients. Because of its granularity, soil consists predominantly of mostly microbially‐generated micro‐gradients that are often extremely steep (e.g. pH gradients formed around cells of a microcolony of microbes producing acid by fermentation of a component of root exudate; oxygen gradients formed by oxygen consumption by the same microcolony). Such gradients are influenced by a multitude of other, mostly microbial, activities, and find themselves within other gradients, sometimes running in different directions, in three dimensions. Because microbial metabolic activities can often proceed at high rates, some gradients can be both extreme and short‐lived, i.e. highly dynamic. Thus, the enormous diversity of environmental conditions characterizing microhabitats resulting from the abiotic properties of soil is hugely amplified by microbial metabolism and the creation of all manner of gradients within the microhabitats. These widely differing and varying microhabitat conditions are a major driver for diversification of microbes and microbial communities in the soil environment, and ultimately also of microbial partnerships with plants and animals. In any case, soil is a pivotally important reservoir of microbial diversity, and provider to practically all life in the biosphere of microbial companions that have profound influences on health and development.
*The granularity of soil allows highly dynamic, multi‐parameter, multi‐dimensional and multi‐directional gradients to form within micro‐niches to create a vast range of discrete micro‐habitats differing one from another in physico‐bio‐chemical characteristics. These in turn select specific, appropriately adapted microbes and microbial consortia, drive microbial evolution and phylogenetic diversification, and steer formation of diverse multilaterally‐interacting and ‐interdependent community associations. The diversity of microbial life in soil exceeds that of all other environments on the planet, so soil is our main reservoir of microbial diversity. Dirt is special, and it is alive*!
  Counteracting the discontinuity of soil are the various life forms that move and grow through it, and water when it saturates, as in flooding episodes, thereby providing some fluid continuity.
  Surface sediments of freshwater and marine bodies are also particulate, granular and heterogeneous, so have some structural similarities to soil, indeed may be considered continuations of terrestrial soil, but importantly they differ by being permanently water‐saturated and hence exhibit a high degree of connectivity and low level of discreteness that tends to dampen gradient formation and reduce the physico‐chemical diversity of microhabitats.
  Of course, the other compartments – air and water – also exhibit heterogeneity and granularity, since they both contain animate and inanimate bodies and particulates (e.g. marine snow, algae, suspended sediment and microplastics in water; dust and moisture droplets in air), that provide surfaces for microbial colonization/habitat niches and can create sharp gradients. But they differ enormously from soil because bulk air and bulk water are to a large extent rather homogeneous and highly connected, and particulates make up only a small fraction of the whole.
10. *The soil microbiome is the essence of soil vitality – a world of innumerable metabolic systems mediating a vast number of chemical reactions – and the motor of soil biosphere services*. Since the appearance of the first forms of life on the planet – bacteria and archaea – 3.8 billion years ago, microbes have evolved to exploit practically anything that can provide energy to drive metabolism and grow, evolutionarily radiating metabolically and phylogenetically in the process to generate the incredible diversity that currently exists, much of which can be found in soil, and that drives its multitude of processes. Soil microbes are master chemists, that carry out a dizzying array of biochemical reactions – so many that, taken together, soil represents the largest chemical reactor on our planet (Ramos and Lansac, 2020; Ramos and Timmis, 2021). Thus far, research has focused heavily on metabolic reactions that mediate the cycling of the major elements of life, i.e. those that are needed to create cells or provide cellular energy: carbon, nitrogen, sulfur, phosphorus, hydrogen, oxygen, etc., and that influence the fate of materials of non‐biological origin humans release into the environment, like polluting pesticides, herbicides and other synthetic organics. But these represent just a tiny fraction of the reactions carried out by soil microbiota, and of the metabolites produced and released into soil, largely to an unknown fate. But, given the importance of biogeochemical cycling of life elements for the health of the biosphere, and for the biodegradation and removal of anthropogenic environmental pollutants, it is not unreasonable to assume that the vast range of reactions thus far unexplored also play a significant role in soil and biota functionalities.
11. *Source of a vast range of biotechnological products and processes*. The exceptional diversity and richness of biochemical capacities found in soil biota, especially microbes, constitutes a treasure trove of organismal and genetic resources for biotechnological applications, including metabolites with nutritional, pharmacological or agrochemical applications, enzymes for green chemistry, plant growth promoting and protecting microbes, pollutant‐degrading and pollutant‐immobilizing microbes and plants, etc. Many of the microbially based applications are key to attainment of the Sustainability Development Goals, as was outlined in a Special Issue of this Journal in 2017 (https://sfamjournals.onlinelibrary.wiley.com/toc/17517915/2017/10/5). Soil microbes are playing and will play central roles in bio‐based economy, the Green Deal (https://ec.europa.eu/commission/presscorner/detail/en/ip_20_1669), and other endeavours to exploit biology for the good of humanity.
*Soil microbes collectively harbour the widest genetic and metabolic diversity on planet Earth and thus are a unique resource for new biotechnological applications, many of which will contribute directly to attainment of Sustainability Development Goals, such as new chemistries aimed at zero greenhouse gas emissions, production‐consumption cycles with zero waste, etc*. (Timmis *et al*., 2017).
12. *Cleansing, recycling and water purification*. Soil is the great cleanser and recycler (*SDGs 6, 12*). Animals mostly do not use all the organic matter in the food they eat and excrete the residuals into and onto soil. Deciduous trees drop their foliage in autumn, and the leaves form soil litter; plants die and fall down; animals on and in soil die; birds drop off their perches; flying insects fall to the ground. Farmers spray fields with pesticides. Some chemical producing factories store/discharge wastes that eventually also percolate into surrounding soils. Microbes tend to bind to soil mineral particles (e.g. Lünsdorf *et al*., 2000), forming biofilms on larger particles, where they access organic matter bound to the particles they colonize and from the interstitial porewater, and metabolize it for growth, thereby purifying the water. Water moves laterally through soil under gravity within a watershed (https://www.pbslearningmedia.org/resource/ket09.sci.ess.water.wshed/what‐is‐a‐watershed/#.X9ePfi1Q1k4), flowing into drainage‐irrigation ditches, streams and rivers to the open sea, or into ponds and lakes, or vertically, to recharge aquifers. While travelling along watersheds and into aquifers, water is subject to physical filtering and purifying microbial metabolic activities that progressively remove pollutants (others may be added during passage along the watershed, of course). Since groundwater is used extensively to supply drinking water, its purification by soil microbes is key to *SDG 6.1*..*achieve universal and equitable access to safe and affordable drinking water for all*.
*Soil microbes, with their exceptional diversity of metabolic activities, use most organic wastes as food and, in so doing, both remove them from soil and recycle them into biomass that enters the food web. They are the great disassemblers, the soil recycling buffer which prevents the accumulation of biological wastes and most pollutants in the environment, and the great cleansers of water transiting through soil to aquifers and surface water bodies*
13. *Climate regulation: role of microbes in greenhouse gas emissions and carbon sequestration* Through the process of photosynthesis in aboveground foliage, plants consume atmospheric CO_2_ and convert it to organic compounds. Excess organic material produced is subsequently transported into the root system, which leaks it into the surrounding soil as exudates. These exudates, and dead plant surface litter, nourish surface and belowground microbes and create hotspots of microbial activity and growth that in turn generates food for other organisms of the soil food web. Consumption of organic C by soil microbes leads to microbial biomass production and CO_2_ (or methane) that is released back to the atmosphere. Although part of the microbial biomass is consumed in the food web, part continues to grow, and part dies and becomes microbial necromass. Microbial necromass constitutes between 30 and 60% of total soil organic carbon in the top layer of soil and is at least one order of magnitude higher than the viable microbial biomass (Glaser *et al*., 2004; Liang *et al*., 2019). Although part of the microbial necromass is used as food by soil organisms, part of it becomes stable organic carbon. This stable – sequestered – organic carbon represents a major fraction of the carbon sink created by plant photosynthesis.
  The fate of newly formed/introduced soil organic carbon – rapid consumption and recycling by soil organisms, or conversion to stabler organic carbon and sequestration – is probably determined by stochastic events: whether or not the first interaction is with consumers or with reactive/absorptive soil minerals, secondary clay minerals and short‐range iron and aluminium phases (Torn *et al*., 1997; Kögel‐Knaber *et al*., 2008; Barre *et al*., 2014; Chen *et al*., 2020a). These latter interactions create complexes characterized by strong chemical bonds that render the organic carbon recalcitrant to biological attack, and physical associations of microaggregates and co‐precipitates that reduce consumer access.
  Peatlands are wetland ecosystems in which permanent waterlogging maintains anaerobic conditions that reduce metabolic rates and favour accumulation of soil carbon (http://www.fao.org/3/a‐an762e.pdf; Loisel *et al*., 2021). They are distributed over the planet, although 80% are in boreal regions of the northern hemisphere. Despite peatlands representing only 3% of the terrestrial surface, they contain about 25% of global C stock, or about twice the amount present in world forests. Global warming is having a major impact on peat reserves: at high latitudes, it is warming permafrost and exposing peat stocks to drying, which then become vulnerable to wildfires. Some of these rage for considerable periods of time (https://www.nature.com/articles/d41586‐020‐02568‐y), releasing large amounts of greenhouse gases that, in turn, reinforce global warming.
  Soil is a carbon sink and the largest reservoir of carbon on the planet. It has been estimated that the top one metre of soil may store up to half of the total planetary C (Lal, 2004). The average age of carbon in soil can be ~ 5000 years old, a period much longer than expected (Shi *et al*., 2020), and one which indicates that C cycling is slower than anticipated (Soong *et al*., 2019). Soil, and the life it harbours, play a vital role in C‐sequestration/C‐emissions and hence global warming (see also Cavicchioli *et al*., 2019).
14. *Soil organic matter: the soil biogeochemical bioreactor*. Recently, Hoffland *et al*. (2020) proposed widening the concept of soil organic carbon (SOC) to soil organic matter (SOM), which more fully describes the soil ecosystem functions and services. In addition to C‐sequestration, SOM encompasses N‐mineralization, aggregation, promotion of plant health and nutrient retention. In this sense, SOM can be considered as a sort of natural bioreactor – one that helps to maintain the function of biogeochemical cycles through a series of biotic and abiotic reactions.
  The sequestration of C necessarily involves a net gain of C in soil; however, a defined interplay takes place in which Hoffland *et al*. (2020) and Valenzuela and Cervantes (2021) proposed that SOM is a heterogenous material driven by microbial decomposition that eventually produces humic substances – a set of high molecular heteropolymers that serve to stabilize organic matter and which possesses redox properties that influence soil biogenic cycles. Interactions between SOM and soil minerals are instrumental in defining soil structure and the formation of microaggregates, pore formation, aeration, water retention and C, N, P, S and Fe metabolism. Humic substances inhibit methanogenesis in anaerobic soils, most likely through the competition between humic acids and methanogens for environmental electrons (Valenzuela and Cervantes, 2021). Humic substances influence the S cycle in soil and promote the oxidation of H_2_S to sulfate, while the flux of electrons in soils also influences the transition between soluble and insoluble forms of iron.
  SOM and SOC have been shown to be important to the healthy growth of plants. They act through suppressing pathogens and providing key nutrients required for growth. For example, SOM can serve as a source of isothiocyanates that are toxic for nematodes. Moreover, SOM may promote the growth of saprophytic microbes that secrete soluble antifungal and bactericidal compounds, as well as volatiles that are involved in inducing systemic resistance in plants (Hammerbacher *et al*., 2019; Vlot *et al*., 2020; Camarena‐Pozos *et al*., 2021). SOM has been proposed to promote plant growth by providing plants with small molecules (i.e. nutrients and factors required for growth), or by promoting the growth of microbes that support N mineralization, P solubilization from phytate or other sources of organic or inorganic P chemicals (Margalef *et al*., 2017; Udaondo et al., 2020). Therefore, the flows of C and other elements in soils are highly dynamic. The interplay between SOM and microbes is so critical that it has been proposed that SOM and microbes together may be judiciously exploited to aid transformation of semiarid soils into fertile soils (Videla *et al*., 2020), and to counteract climate change‐promoted desertification.
  Strategies that aim to preserve and build healthy soil must consider that soil is a dynamic system and needs to be treated holistically to preserve the set of complex interplay and equilibria between physico‐chemical and biological characteristics.
*Soil provides a multitude of goods and services that underpin ecosystem functioning, and are vital to human survival and wellbeing*. *Microbes play key roles in the provision of soil ecosystem services. It is crucial to conserve global soil resources and maintain them in a healthy condition*.

## Higher organism life is all about microbiomes: holobionts

Essentially all (if not all) animals and plants of the biosphere are covered in diverse microbes which collectively make up their microbiomes. Microbiomes provide their hosts with essential services, such as digestion of food materials, provision of essential vitamins, protection from pathogens, and confer upon hosts diverse behavioural traits. In return, the host provides its microbiome with food and privileged habitats subjected to fewer stresses and less competition than non‐microbiome habitats. The host plus its microbiome is designated the holobiont. The ecophysiological behaviour and identity of one partner of the holobiont is determined to a significant extent by the attributes of the other. The host and microbiome co‐evolve over time and in response to short‐ and long‐term selective pressures. The genetic resources available for adaptation and evolution consist not only of the 10,000 to 100,000 plant genes, but also of the vastly more microbiome genes.

The microbiome is an essential component of the holobiont: most plants and animals lacking a microbiome are inviable in nature. Microbial life without higher organisms is possible, just consider the first 1.8 −2.3 b years of life on the planet, cooling fresh lava from a volcanic eruption which is first colonized by microbes, life in the deep subsurface such as the Iberian Pyrite Belt (Puente‐Sanchez *et al*., 2018), hypersaline brines (van der Wielen *et al*., 2005), etc. But not the other way round.

The intimate and comprehensive interactions between host and microbiome, and between holobiont and environment, most of which we have yet to identify and characterize, are highly integrated and fine‐tuned, responding to changes in a coordinated manner to maximize benefit/minimize damage. If such interactions become perturbed, all partners – the plant/animal, the microbiome, the environment = soil – can suffer, and the resilience of the holobiont system to stresses is lowered.

Plant microbiomes consist of surface microbes and microbes inside plant organs, tissues and cells. The global surface area of plant leaves has been estimated to amount to > 6 x 10^8^ km^2^, housing some 10^26^ microbial cells, which represents one of the largest microbial habitats on Earth (Morris and Kinkel, 2002). And the rhizosphere (McNear, 2013), the root:soil interface – the surface of plant roots and soil intimately associated with and directly influenced by roots – harbours 10^8^‐10^12^ microbes per gram. Within their tissues, plants contain ca. 10^7^ microbes per g of root, 10^3^ −10^4^ per g leaves, and 10^2^ per g of seeds or flowers (e.g. Abdelfattah *et al*., 2021; Compant *et al*., 2021). Different plants recruit different microbes, thereby assembling a wide diversity of holobiont compositions that provide considerable capacity for adaptation‐evolution of the holobiont to changing environmental conditions and stresses, and for exploration of new habitats to colonize (de Zelicourt *et al*., 2013). The plant:microbe partnership that constitutes the plant holobiont provides reproductive continuity of the microbial consortia of the habitats. Microbes drive plant diversity (van den Heijden *et al*., 2008; *SDG15*).

Ecto‐ and endosymbionts of plant roots play a key role in the acquisition of otherwise non‐bioavailable elements and minerals essential to plant growth through, for example, fixing atmospheric nitrogen and providing it as ammonia, solubilizing phosphate, iron, etc. In degraded and stressful environments, the microbiome can buffer the stressors enabling plants to grow on otherwise inhospitable soils and to grow faster in challenging environments (de Zelicourt *et al*., 2013). For example, some members including endosymbionts can increase plant tolerance of stressors like aridity and coldness, thereby extending the range of habitats available to plant growth and hence the geographical range of colonization/cropping. Conversely, plants can buffer stressors affecting microbes, enabling a more diverse range of microbes to propagate; for example, in arid zones, plant roots can seek out water and provide it to their microbial partners.

On the other hand, the microbial partners, in occupying plant surfaces, reduce the colonization options for pathogens and, in some cases, produce antibiotic substances that inhibit pathogens. Also, endophytic microbes can counteract infections: for example, fungal attack of plant roots was shown to trigger enrichment of a specific antibiotic‐producing endophytic bacterial consortium. Inactivation of the specific antibiotic eliminated the protective effect, and transfer of the endophytic bacterial consortium transferred the protective effect (Carrion *et al*., 2019).

Moreover, microbes orchestrate aspects of Induced Systemic Resistance (ISR), plant resistance responses to pathogen and insect herbivore attack (van Peer *et al*., 1991; Pieterse *et al*., 2014; Vlot *et al*., 2020). Two volatile plant defence hormones, methyl salicylate and methyl jasmonate, systemically induce defence responses, such as the production of antimicrobial phytoalexins at the site of infection and in plant parts distant from this, without the necessity of having a signal transit through the vascular system. The trigger for enhanced production of these hormones are a number of ISR elicitors that include, among others, microbially produced antibiotics, such as 2,4‐diacetylphloroglucinol and pyocyanin; flagella; *N*‐acyl homoserine lactones; iron‐regulated siderophores; biosurfactants and volatile organic compounds, such as 2R,3R‐butanediol or medium chain fatty acids. The ISR response also involves the emission of volatiles by plants that mediate interplant communication by signalling information about imminent dangers.

## Soil is the most important microbiome reservoir

Good health in the animal (including human) and plant holobiont is highly correlated with a high diversity of their microbiomes. Unhealthy holobionts often have a less diverse microbiome than healthy ones, a condition expressed as microbiome dysbiosis (though, as yet, this term has no clear definition; Brüssow, 2020). A hint of the relevance of this is the finding that children growing up on farms, i.e. environments with high microbial loads and diversity, experience lower levels of allergies than their peers in urban settings, i.e. environments with relatively poor microbial loads and diversity, which is assumed to reflect the higher diversity of their microbiomes and the importance of this for the microbial steering of development of a healthy immune system (Ege *et al*., 2011; Stein *et al*., 2016; Finlay and Arrieta, 2016; Gilbert *et al*., 2017; Mezouar *et al*., 2018).

Soil houses the greatest diversity of microbes and hence acts as the most important diversity reservoir for seeding microbiomes – of plants, animals and us – and hence for organismal health (*SDGs 3, 15*). Moreover, the soil microbial diversity reservoir continues to enrich microbiomes throughout holobiont life, and to replenish diversity lost from all manner of animals and plants through, for example, disease or other stresses and, additionally in humans, through clinical treatment with antibiotics and other antimicrobial agents. Maintenance of microbiome diversity in animals, including humans, occurs by direct contact with soil, but also via food, the soil‐originating microbiomes of raw salads/vegetables, grains and fruits of which provide constant microbial diversity input into our microbiomes. The rhyme ‘An apple a day, keeps the doctor away’ is not only an issue of nutrients, fibre and physical action on teeth of apple flesh, but also one of enrichment of our microbiomes (Wassermann *et al*., 2019).

*Soil is a major microbiome‐provider and renewer of higher organisms in the biosphere*

## The soil biome is highly networked (‘*The soil is the great connector of our lives, the source and destination of all’*. Berry, 1977)

Though we have emphasized the fact that the abiotic components of soil are discontinuous, and the discrete microniches they create are major drivers of microbial diversity, the intimate connections between host and microbiome, and between holobiont and environment are complemented by *bioconnectivity* within the soil system, which endows the soil biome with biocohesion and bionetworking over considerable distances. This connectivity consists of different components.

1. *Physical bioconnectivity – water and air*. While drier soils contain a myriad of discrete niches with different environmental conditions, increasing water content caused by rain, flooding, etc., provides physical connectivity between niches, lowers the steepness of gradients formed when soils were drier, and renders soil niches less heterogeneous, resulting in less diversity granularity. On the other hand, water connectivity also promotes migration of microbes through soil, and thereby increases the potential for reassortment, creation of fitter, and adoption of new evolutionary trajectories, of microbial communities when soil dries out. Persistent flooding caused by extreme weather events creates longer term soil connectivity and decreased diversity, and also may result in upper horizons becoming anaerobic and thus induce a shift from aerobic to anaerobic communities (Azua‐Bustos *et al*., 2018; Mestre and Höfer, 2020). And, extreme regular (e.g. Nile) and stochastic flooding events mediate mass redistribution of soil materials and their biota.
  Air connectivity is of two types. The aboveground atmosphere directly influences surface microbiota through the carriage of microbes via wind. This is amplified by mass redistributions of soils and biota by hurricanes and dust storm events (Sanchez de la Campa *et al*., 2013; https://public.wmo.int/en/our‐mandate/focus‐areas/environment/SDS; https://earthdata.nasa.gov/learn/sensing‐our‐planet/saharan‐dust‐versus‐atlantic‐hurricanes; https://www.youtube.com/watch?v=ygulQJoIe2Y&feature=youtu.be; https://www.theatlantic.com/photo/2018/03/the‐strange‐beauty‐of‐sandstorms/556607/). Surface‐deposited biota may then move down into the soil body carried by percolating water or other mechanisms. Belowground air in soil pores and channels may be in part static, in part moving in response to changes in soil humidity and local air pressures caused by biota movements, etc. Soil pore and channel air enables diffusive movement of gases and gaseous metabolite connectivity between communities, and thus is essential to the functioning of the biogeochemical cycles. This is exemplified by pore filling by water during waterlogging events and the resulting restriction of diffusion of gases, like oxygen, causing a switch from aerobic metabolism/communities to anaerobic metabolism/communities. The air in soil pores also is a conduit for diffusion of volatiles, especially signalling volatiles that mediate chemical connectivity and dialogue between diverse members of the soil biota (see below).
2. *Biological connectivity*. The soil microbiome is an intimately networked living mesh connecting diverse forms of life and non‐life of the soil matrix (see e.g. Fierer, 2017). Some microbes can swim or glide, if there is a film of water on the substratum they occupy, and hence move around within a relatively limited space to explore it for better habitats. In so doing, they may transport non‐motile microbes, or at least their spores, sometimes over cm distances (Muok *et al*., 2021) Importantly, filamentous growth allows microbes like fungi and actinomycetes to seek, penetrate and explore new niches over considerable distances. In so doing, they carry non‐filamentous microbes along with them on their surfaces, and provide surfaces for motile microbes to swim along – the so‐called fungal highways – thereby creating and maintaining bioconnectivity and its metabolic and ecophysiological activities (e.g. Pion *et al*., 2013). Such highways are not simply random routes to somewhere/anywhere: they are at least partly created in response to signals detected by sensory systems of the highway engineers, i.e. via chemotactic responses, and used by them and their microbial passengers for targeted directional travel to new sources of food, etc. (Furano *et al*., 2010). Filamentous microbes are the microbial equivalent of the soil engineering earthworms, moving through the soil, seeking and sharing manna, and spreading diversity.
  While some non‐filamentous microbes disperse via filamentous microbes, others disperse via the surfaces of plant roots (like sitting on the top of an open‐roof bus), which also seek, penetrate and explore new niches. Some microbes, like the mycorrhizal fungi, form specific and intimate associations with plant roots, becoming part of the root itself and growing with it. Yet other microbes invade plant roots and become endosymbionts, thereby travelling around but always sitting inside the bus. Until the root becomes injured, when they are ejected from the bus, after which they must wait for the next one to come along. During these journeys, microorganisms exchange DNA with others, sometimes with relatives, sometimes with strangers, by transformation, transduction or conjugation. Analysis of the genomes of soil microorganisms reveals that their genomes are mosaics, reflecting extensive gene exchange and flow, and confirming the evolutionary dynamism and opportunism of soil‐dwelling microbes.
  And of course, microbes disperse, passively and actively on all other forms of life in soils. Thus, despite the facts that the majority of microbes in the soil microbiome typically have sizes of < 1 micron–a few microns in length and breadth, and live in a matrix that is highly discontinuous, they are nevertheless highly interconnected through the long‐distance filamentous growth of filamentous microbes and plant roots, and the burrowing lifestyles of soil animals. Given that the largest organism thus far found on the planet is a soil‐dwelling microbe, the fungus *Armillaria ostoyae*, which spreads over an area of 2,385 acres (https://www.youtube.com/watch?v=vWAA‐SrrFUQ), it is interesting to speculate on the length of its fungal highways, how many other microbes it has thus far ferried through soil, and over what distances!
3. *Electrical bioconnectivity*. One of the most exciting recent findings is the existance and roles of nanowire and cable bacteria, which conduct electrons over considerable distances to electron acceptors in soil, muds and sediments, and thereby create electrical connectivity. Soil bacteria producing nanowire structures include *Geobacter*, *Shewanella* and thermophilic bacteria. Different kinds of conductive appendages have been described (Lovley and Waker, 2019). Cable bacteria form filaments in marine and freshwater sediments that connect ecophysiologically distinct oxic and reductive habitats separated by considerable distances (Kjeldsen *et al*., 2019), and control *inter alia* iron and phosphorous dynamics, which influence eutrophication (Sulu‐Gambari *et al*., 2016).
4. *Chemical bioconnectivity*. Supporting networks of physical and organismal bioconnectivity are other networks, the principal one being chemical in nature. Chemical networks in soil are generally poorly understood, due to the technical challenges posed by the system, but are highly important. Probably the most pervasive form of chemical connectivity in the microbiome is localized metabolite exchange among the different members of microbial communities occupying soil niches (Fritts *et al*., 2021), which evolve for maximal resource utilization efficiency, and which thereby structures the community composition (Pelz *et al*., 1999). This chemical bioconnectivity may be primarily microbial – within microbial communities – or also involve higher organisms.
  Siderophores, diverse biochemicals that capture iron – a co‐factor of many cellular proteins and a poorly soluble, poorly bioavailable metal limiting microbial growth – and ferry it into the cell, are another class of chemicals whose production and uptake specificities constitute a complex chemical connectivity in the soil microbiome.

Another well‐studied example of chemical connectivity is signalling. Plants deposit 10‐40% of the photosynthetically fixed carbon via their root system in soil (Compant *et al*., 2021). As a consequence, the microbial density in the rhizosphere is 10‐fold to 100‐fold higher than surrounding soil and the composition of its microbial community is distinct from that of the surrounding bulk soil, a phenomenon called the rhizosphere effect (Walters *et al*., 2018; Kong, *et al*., 2019). For this kind of successful mutualistic association, host plants and microbes have co‐evolved for 700 million years and have learned to respond to reciprocal signals and prioritize their responses to develop a lifestyle that provides optimal mutual benefits. In the well‐studied rhizobial symbiosis, host‐secreted strigolactones and flavonoids stimulate the production of symbiotic Sym and Nod factors by the microbes, which in turn activate a common symbiosis (Sym) signalling pathway in plant roots that leads to establishment of a successful symbiotic relationship (Jimenez‐Guerrero *et al*., 2021). As the reservoir from which rhizosphere inhabitants are selected, soil type is an important factor in determining rhizosphere microbial community composition (Walters *et al*., 2018). Nonetheless, in the same soil, different plant species select distinct microbial communities through differences in the composition of their root exudates.

One of the most important signalling systems is that of so‐called quorum sensing (QS), which is a cell‐to‐cell chemical communication mediating ecophysiological responses calibrated to population density. QS is based on the production and detection of, and response to, small molecules (homoserine lactones, quinolones, small peptides, etc.) called autoinducers. Threshold population densities produce threshold levels of autoinducer concentrations that trigger synchronous gene expression in the population leading to new ecophysiological responses. Many of these are involved in the ‘ecological war’ for colonization of a niche and require high cell numbers for success (Mukherjee and Bassler, 2019), such as biofilm formation – self‐crafted, protective microbial habitats on surface substrata, switching from a saprophytic to a parasitic lifestyle, competence for DNA uptake, secondary metabolite production, etc. At the community level, autoinducer molecules modulate gene expression at the intra‐clonal, intra‐species, intra‐genus, inter‐species and inter‐genera, and may even alter host expression in the holobiont.

Microbial volatiles are also important chemical signals, which readily disperse in the pore space air and act over considerable distances (Weisskopf *et al*., 2021). We are all familiar with pleasant earth‐odours we detect through our olfactory receptors after rain, one of which comes from geosmin, a compound produced by soil *Streptomyces*, cyanobacteria and some fungi. In fact, not only we smell it, but arthropods also do and also seem to find it pleasant. Geosmin biosynthesis is part of the complex set of responses of *Streptomyces* to nutrient depletion that set in motion the process of sporulation. As a result, *Streptomyces* sporulates and produces geosmin which attracts soil‐dwelling arthropods. These feed on the *Streptomyces* colonies, with spores being ingested and attaching to the antenna of the arthropods, which then move on and distribute the spores to new, potentially nutrient‐rich habitats (Becher *et al*., 2020). This attraction of *Streptomyces* of arthropods is specific, as insects and arachnids do not respond to geosmin.

Signalling between soil organisms via volatiles is pervasive (Audrain *et al*., 2015; Schulz‐Bohm *et al*., 2017; Tyc *et al*., 2017; Hammerbacher *et al*., 2019; Schenkel *et al*., 2019; Camarena‐Pozos *et al*., 2021; Weisskopf, *et al*., 2021). However, soil compaction through use of heavy machinery, and flooding events, reduce air spaces and channels in soil, and hence reduce the diffusive flow of signalling volatiles. The consequences are reduced communication between plants and a local build‐up of the signalling molecules around the producing plant. The local build‐up of ethylene in compacted soil has been shown to reduce root growth of the producer and hence exploration of soil for nutrients and water, with the resulting negative impact on plant growth (Pandey *et al*., 2021). Ethylene has been proposed to be an early warning signal to roots to avoid compacted regions of soils (Pandey *et al*., 2021).

So: soil and its biota are, despite soil heterogeneity and granularity, effectively networked. In fact, connectivity at all scales is a characteristic of biological systems (e.g. see Timmis, 2018). Connectivity is a crucial feature of healthy soils because it allows communication over distance in mostly patchy, discontinuous habitats, and facilitates maintenance of diversity and adaptation and optimization of community life to the terroir.

*Connectivity is a major contributor to the services soils provide and to soil resilience. Most importantly, it is an enabler of coordinated bionetwork action:reaction, i.e. responsiveness to changes in environmental conditions, either to profit from favourable opportunities or to mount defensive actions/restorative responses to protect against detrimental changes, such as stresses, shocks, hazards and abuses*.

The connectivity of soils exists at all scales, from the microscale (e.g. metabolite exchanges between microbes), the cm scale (e.g. microbe distribution by fungal hyphae), the metre scale (e.g. microbe distribution by plant roots, earthworms and burrowing moles), kilometre scale (e.g. mass redistribution of soils and biota by regular – e.g. Nile – and stochastic flooding) and 10s to 1000s of kilometre scales (e.g. via hurricanes and dust storm events (https://public.wmo.int/en/our‐mandate/focus‐areas/environment/SDS; https://earthdata.nasa.gov/learn/sensing‐our‐planet/saharan‐dust‐versus‐atlantic‐hurricanes; https://www.youtube.com/watch?v=ygulQJoIe2Y&feature=youtu.be). However, despite the fact that connectivity brings major benefits, it has a downside in that seriously prejudicial events in one region can be transmitted to neighbouring regions, triggering domino effects/tipping points that can lead to system collapse (e.g. Hernandez *et al*., 2021).

## Soils are alive but many are unhealthy and experiencing reducing fertility, others are dying, and most are suffering erosion. (‘*Soil erosion is as old as agriculture. It began when the first heavy rain struck the first furrow turned by a crude implement of tillage in the hands of prehistoric man. It has been going on ever since, wherever man’s culture of the earth has bared the soil to rain and wind’*. Hugh H. Bennett and W. C. Lowdermilk, ~ 1930s).

Many soils are unhealthy, and some are being permanently lost through erosion. While the transition of healthy to unhealthy soils, and soil loss, can occur through natural causes, much is due to abuse through human practices ‐ primarily overexploitation of its resources/services ‐ that leads to soil degradation.

1. *Soil: the vital disappearing resource: desertification and erosion*. As indicated above, it takes a century to build three millimetres of topsoil. However, the rate of production of soil is dwarfed by its rate of loss through erosion – wind blows it into the air, some of it descending into surface water bodies, and rain and especially flooding events wash it into rivers and thence to lakes and oceans. Soil erosion not only leads to losses, but its subsequent deposition in water bodies often causes waterway clogging that itself may increase flooding events, and flow and mixing reduction, thereby creating stagnant water bodies and loss of aquatic species. And, most importantly, once in aquatic systems, fixed carbon becomes more accessible to microbial metabolism and emission as greenhouse gases.
  Drylands (https://www.carbonbrief.org/explainer‐desertification‐and‐the‐role‐of‐climate‐change), i.e. lands receiving too little water to maintain healthy soils, constitute more than 40% of the world’s terrestrial surface, include 45% of the world’s agricultural land (Burrell *et al*., 2020) and are home to around 2.7 billion people. Drylands are particularly susceptible to degradation through natural and human activities, which in turn can lead to desertification (https://www.ipcc.ch/site/assets/uploads/2019/08/2d.‐Chapter‐3_FINAL.pdf. A soil is considered to become desertified when it no longer supports the same plant growth it had in the past and the change is permanent on a human time scale. The United Nations has reported that around 33% of all land that is not covered by snow is experiencing desertification. Desertification affects 169 countries and 1.5 billion people. Desertified land is particularly susceptible to erosion.
  Human activities that contribute to desertification through degradation of land in areas with low or variable rainfall, include mining and farming/ranching (clearing land of natural vegetation, soil compaction by machines and animals (e.g. Troldborg *et al*., 2013; Burrell *et al*., 2020; Krauss *et al*., 2020), soil tilling, crop depletion of soil nutrients, etc.), lowering of water tables through excessive extraction of groundwater, etc.
  Global warming and extreme weather events can, on one hand, cause increased drying of drylands, which become even more vulnerable to soil losses by wind erosion. On the other hand, storms of increasing frequency and violence, while bringing rain, cause increased soil losses by water erosion (Burrell *et al*., 2020). It has been estimated that half of the topsoil of the planet has been lost during the last 150 years (https://www.worldwildlife.org/threats/soil‐erosion‐and‐degradation). Expressed another way, there is an annual loss of 75 billion tons of fertile soil and 12 million hectares of productive agricultural land that could otherwise produce 20 million tons of grain (*SDG 2, 3*) and represents lost earnings of USD 42 billion (*SDGs 1, 8*). (www.unccd.int/actions/united‐nations‐decade‐deserts‐2010‐2020‐and‐fight‐against‐desertification). The result of desertification is a reduction in essential services provided by soil, poverty, starvation and uncontrolled movement of desperate people seeking more secure places to live **(** *SDGs 1, 2, 3, 4, 10, 11, 13, 15, 16, 17*).
2. *Soil: the vital disappearing resource: soil sealing*. An enormous area of healthy soil providing vital services has been, and continues to be covered with impermeable materials, like concrete and bitumen, to expand urbanization and create infrastructure – dwellings, premises for business, etc., roads, carparks, airports, shopping centres, and so forth. Economic stimuli following downturns will often favour infrastructure projects, like new highways. Urban renewal often involves covering over brownfields containing legacy pollutants to create more dwellings/premises and infrastructure, rather than remediating the contaminated sites: an out‐of‐sight, out‐of‐mind response to the choice of extracting profit through creation of buildings or investing to create healthy soils. Humankind has developed a concrete addiction.
  But sealing soil eliminates its vitality and the services it provides the biosphere: there is little or no carbon input from plants, gas exchange permitting biogeochemical cycling, or water input essential for life of biota. Soil is suffocated to death. Urban areas are starved of soil system services, including uptake of CO_2_, which is produced in vast quantities by urban activities. Moreover, sealing eliminates permeable surfaces that otherwise would mitigate flooding events, through the water uptake capacity of underlying soil and its ability to accommodate run‐off during episodes of heavy precipitation. This is particularly acute for built‐upon floodplains, especially where river engineering/straightening has been carried out.
3. *Phosphorus: the diminishing vital component of soil fertility*. Soil fertility is defined as ‘*the ability of a soil to sustain plant growth by providing essential plant nutrients and favorable chemical, physical and biological characteristics as a habitat for plant growth’*. (http://www.fao.org/global‐soil‐partnership/areas‐of‐work/soil‐fertility/en/). The primary limiting nutrients in most soils are nitrogen and phosphorus. Whereas there is an unlimited supply of atmospheric nitrogen available for chemical and biological fixation to biologically useable forms, global sources of phosphorus are limited geological deposits, located primarily in Morocco and the Western Sahara, that are rapidly depleting (Cordell *et al*., 2009; https://www.phosphorusplatform.eu/images/download/HCSS_17_12_12_Phosphate.pdf). Despite processes that recover P from wastewater, etc., for recycling, the fate of the majority of mined P consists of a linear pathway of exploitation: mining > agricultural use > leaching to surface freshwater > transfer to marine waters. This is unsustainable (Carpenter and Bennett, 2011). Importantly, P in soil (i.e. intrinsic P, plus any P added as fertilizer), and hence soil fertility, is being lost not only by leaching to surface waters but also by soil erosion (Alewell *et al*., 2020). Paradoxically, despite the universal scarcity of P in soils, and the diminishing availability of mined P, its flow into surface freshwaters exceeds planetary boundaries – upper tolerable limits – for eutrophication (Carpenter and Bennett, 2011), which clearly testifies to the unsustainability of current practices for use of P. Added to this is the fact that the toxic metal cadmium is a component of phosphate rock, so annual applications of P fertilizer to agricultural soils result in their progressive contamination with cadmium (https://www.phosphorusplatform.eu/images/download/HCSS_17_12_12_Phosphate.pdf). The phosphate rock from Morocco has particularly high cadmium levels, so this will worsen in future, unless steps are taken to seriously reduce P fertilization of agricultural soils.
  It is absolutely essential to transit from a linear pathway agriculture to a circular agriculture (e.g. Rhodes, 2017), analogous to the circular economy, with minimal inputs and wastes, and recycling of wastes. And not only with regard to phosphorus fertilizers.
4. *The demand for an ever‐increasing supply of food through agriculture is a key contributor to soil loss. ‘We abuse land because we regard it as a commodity belonging to us. When we see land as a community to which we belong, we may begin to use it with love and respect’*. *(*Aldo Leopold, A Sand County Almanac, 1949). (Expressed another way: if we perceive soil as a living entity that is a major contributor to our wellbeing, and hence a personal friend, we may begin to abuse it less.) The solutions to feeding the expanding population thus far explored have emphasized the intensification of farming and the enlarging of agricultural acreage through deforestation. Neither of these are sustainable, especially in the context of a continuously increasing world population and the currently unfolding disaster of global warming.
  - Fertilizers – eutrophication and toxic surface waters. Plant growth, and hence agricultural crop yields, is mostly limited by bioavailable nitrogen and phosphorus in soil. Intensification of farming *inter alia* involves alleviation of these bottlenecks through supplementation of nitrogen and phosphorus nutrients. However, fertilization with chemical nutrients distorts the natural soil chemistry and biogeochemical cycles, and many studies in different parts of the world have shown that they reduce soil biodiversity, and hence the natural ecophysiological networks and food webs. This is not the case when soils are fertilized with organic materials, like stubble, compost and biochar (e.g. Chew *et al*., 2020; Spanoghe *et al*., 2020).
    Moreover, only a fraction of the chemical nutrient applied is used by the target crop plants themselves; the remainder washes out and migrates through the watershed to surface water bodies where it promotes the growth of other photosynthetic organisms, cyanobacteria and dinoflagellates (some of which produce toxins), and causes impacted water bodies to become eutrophic. Blooms of toxic cyanobacteria and dinoflagellates perturb normal food webs, often creating much biomass that is not grazed but instead dies and is degraded by heterotrophic microbes that deplete oxygen levels, thereby killing off fish and much of the other animal life. Particularly in coastal systems, this can lead to a reduction in finfish stocks and the inability to market toxin‐contaminated shellfish stocks.
*The use of agrochemical fertilizers to increase food yields on terrestrial farms reduces soil microbial diversity, leads to eutrophication of aquatic systems, and can significantly reduce food fish yields, thereby off‐setting some of the food production gains*.
  - Intensive farming practices can perturb and reduce microbial diversity and soil health. Plant diseases and pests result in 20‐30% losses in crops; monocrops grown to high density are particularly susceptible to such losses. The standard solution adopted to reduce this problem is the application of pesticides. Pesticides pollute soil and reduce its biodiversity. The planting of monocrops significantly reduces soil biodiversity. Importantly, regular disturbance of surface soils through tilling and use of heavy machinery, stubble burning, overgrazing, etc., fragments the soil‐stabilizing polymer glues produced by surface algae and cyanobacteria, thereby destroying soil structure and producing soils from which water evaporates more rapidly, and reduces deposition of stable carbon (Krauss *et al*., 2020). Such soils are more susceptible to degradation, and wind and water erosion.
    Overwatering, through automatic sprinklers and irrigation, washes out nutrients and can reduce diversity, also increasing the vulnerability of soil to erosive forces.
    Demands for increased food production (and agricultural profits) through intensification of farming often means using the same land 2 or even 3 times each season: the soil and its biota are subjected to the stresses of intensive practices several times over short intervals, with little time to recover. Earlier practices of growing only one crop per season, and leaving fields fallow for a year every three years, have by choice or necessity become a rarity.
*Efforts to increase agricultural productivity involves farming practices that overexploit the capacities of soils, reduce the diversity of their biota, render them less resilient‐more susceptible to erosion, and ultimately result in loss of soil essential for plant growth*.
  - Deforestation: fool’s gold. Another approach to increasing food production is to increase the availability of agricultural land, typically by deforestation (http://www.fao.org/state‐of‐forests/en/; https://ec.europa.eu/environment/archives/greenweek2008/sources/pres/3_8_Achard.pdf While this does indeed boost available acreage of cropland, it removes ancient forests. As old forests dwindle in area, their ability to provide new farmland decreases, so this strategy to increase food production to supply a growing world population is clearly unrealistic and unsustainable.
    - *Acceleration of global soil losses*. Most importantly, the provision of new agricultural soil by deforestation not only cannot keep up with losses of existing agricultural land, but also makes previously stable soils of old forests susceptible to erosion, because deep and permanent tree roots, the understory (the vegetation under the canopy between the trees) and tree litter retain soil better than shallow crop roots present for short growing seasons.
    - *Release of fixed carbon and reduction in the global carbon sink*. Plants live by photosynthesis – the conversion of sunlight and carbon dioxide in the atmosphere to sugars and hence energy – and thus draw down and are a sink for the important greenhouse gas carbon dioxide. Old forests are not only global sinks for atmospheric CO_2_ and hence buffers against global warming, but also major reservoirs of fixed carbon. Deforestation by slash and burn is thus doubly detrimental to climate change: it releases carbon that has been fixed for decades/centuries and concomitantly eliminates a major CO_2_ sink.
    - *Loss of biodiversity and disruption of ecological networks* Deforestation destroys natural habitats and leads to habitat fragmentation, which in turn perturbs ecological networks and has a lasting impact on Earth’s ecosystems (Haddad *et al*., 2015). Rainforests in particular are also important reservoirs of biodiversity, including microbial diversity, and reductions in their sizes leads to loss of unique biodiversity, accelerating extinctions of endangered species.^1^
*Ancient forests are special: they are unique elements of biosphere heritage, habouring unique biodiversity and substantial amounts of sequestered carbon, and stabilising and nurturing precious soils. It is likely that deforestation will come to be regarded in future as a biosphere ecocrime*.
  - Drainage of wetlands and peatlands. Like ancient forests, wetlands and ancient peatlands represent opportunities to increase agricultural land, namely by draining (Loisel *et al*., 2021). However, both provide important ecosystem services (http://142.44.210.7/bitstream/123456789/143/1/Millennium%20Ecosystem%20Assessment.%20ECOSYSTEMS%20AND%20HUMAN%20WELL‐BEING%20WETLANDS%20AND%20WATER%20Synthesi.pdf; Kimmel and Mander, 2010). Peat is also a commodity: it is used as a soil substitute and amendment for garden soil, and as a filtration medium and absorbant in industry, so is extracted from peatlands for commercial purposes. Drainage and extraction promote fixed carbon losses and reduce the stability of previously stable peatlands and carbon stocks.
5. *Soil ill health caused by global warming (SDG13)*. Global warming caused by anthropogenic activities has diverse effects on soil health. The direct effect of warming soils influences their microbiomes, though as yet it is unclear what consequences these changes will have for rhizosphere and bulk soil microbes. Similarly, plants will be subjected to higher ambient temperatures, which will influence their physiology and that of their microbiomes. Again, the consequences of these changes for plant holobionts and their all‐important organic carbon inputs into soil are unclear.

In any case, soil warming generally increases soil water loss rates, thereby facilitating the process of desertification, reducing the ability of the soil to support plant growth, and reducing resilience to erosive forces. It has been calculated that a 2°C increase in temperature would result in aridification of 30% of the total land surface of the planet. In forest areas, global warming may create aridity and the drying of vegetation which increase their susceptibility to wildfires. In regions that regularly experience wildfires, global warming extends the fire seasons. In addition to destroying vegetation and releasing its carbon to the atmosphere, wildfires have two important effects on soil. On one hand, they create considerable quantities of pyrolysed organic matter, which constitutes stable soil beneficial carbon (González‐Pérez *et al*., 2004). On the other hand, they produce ‘hydrophobic soils’ by volatilizing lipid and wax materials in soil which then condense in upper soil layers. Hydrophobic soils are less able to soak up water and thus more susceptible to erosive forces (Certini, 2005). Slash and burn practices have similar effects on soil.

Of course, global warming‐triggered extreme weather events, like storm‐ and hurricane‐associated high winds and extreme precipitation, are extremely erosive and lead to major soil losses. Global warming also promotes the melting of glaciers and polar ice sheets which engenders rising sea levels, which in turn submerge low‐lying islands and atolls and erode coastlines, thereby reducing available land. And extreme weather events result in coastal inundations and flooding, and hence salinization of wetlands and agricultural land. Salinization of wetlands significantly impacts their biota and the biogeochemical activities and other ecosystms services they provide (Herbert *et al*., 2015). And, since some crops, like maize, potatoes, field beans, are sensitive to increased salinity, coastal flooding renders farmland less versatile for agricultural purposes (e.g. Gould *et al*., 2020).

*Current degradation and losses of topsoil constitute one of the major Grand Challenges of our times. Soil losses are losses of life that catalyses the multitude of processes which sustain life on the planet*.

## Reversing the deterioration of soil vitality and loss, and restoration of health: plant–microbe holobionts to the rescue! (‘*We know more about the movement of celestial bodies than about the soil underfoot’*. Leonardo DaVinci, ~ 1500)

Soil health is crucial to soil retention, plant health, agricultural productivity, biodiversity conservation, and the host of other services healthy soils provide. Stresses and abuses that degrade its vitality, fertility and resilience make it vulnerable to erosion. Many soils around the world are sick and dying, dying at an alarming rate. They are dying because of a pandemic, and the pandemic agent is the human race. But: if we are the problem, we are also able to identify and implement remedies. However, we need to consider the issue as a health/wellness issue, a global health issue, needing the classical medical approaches of diagnosis‐prevention‐treatment pathways, and definitely not as a commercial resource issue. Importantly, we also need to create an integrated socio‐economic‐political‐legislative framework – nationally and internationally – for soil resource protection and management. And, crucially, we need to see the soil crisis as one component of a series of interconnected local, regional and global crises representing Grand Challenges that need to be addressed simultaneously in a coherent manner.

*Plants may be viewed as a global mining operation that extracts CO_2_ from the atmosphere, refines it to energy‐rich sugars, and delivers it via its vascular conveyer belt to belowground microbes. This in turn fuels the massive belowground microbial metabolic activity and biomass production, some of which ends up as the all‐important fixed carbon/humic substances, that are essential elements of healthy soils. The ‘4 per 1000’ initiative (*Chabbi *et al*., 2017 *), which aims to increase SOC by 4/1000 annually, is an important goal aimed at mitigating climate change, and improving soil quality and food security*.

We urgently need to adopt a systems approach to soil health, integrating basic ecological principles that operate at all levels and scales, from the scale of the plot to the scale of the landscape, integrating the uncultivated areas, so that territory, producers, consumers and stakeholders are constructively engaged and learn to appreciate that a greater biodiversity is a key aim towards attaining better health. Healthy soils have a long‐term diverse plant/crop cover (e.g. Garland *et al*., 2021) that, through their surface cover and root systems that hold soil masses together, provides both protection against erosion and a significant input of carbon that feeds a highly diverse soil microbiota. This, in turn, provides the plants with goods and services that are important for their health, on one hand, and constitutes a key element of a diverse soil food web and the agents of soil chemistry and the biogeochemical cycles, on the other (e.g. de Vries et al 2013).

But we only have fragmentary information and poor understanding of key processes underpinning soil health and its deterioration so, in addition to setting in motion necessary things we are sure of/accelerating activities already in motion, we must in parallel carry out the research needed to provide fundamental information and understanding we are lacking.

1. *The plants: a focus on soil beneficial properties*. (‘*Manners maketh man’* William of Wykeham, Bishop of Winchester (14^th^ Ct), and '*Microbiome maketh plant'* Timmis and Ramos, this Editorial). Thus far, plant and soil research has mostly focused on commercial interests: the discovery and creation by breeding/genetic manipulation of plants exhibiting higher yields, longer shelf lives, different/intenser flower colours and scents, and so forth, all in the context of monocrops. However, breeding primarily for traits of commercial interest has often resulted in increased plant health vulnerabilities. This is nicely illustrated by a recent study of the tomato: increasing targeted evolution of the tomato has resulted in increasing susceptibility to fungal attack and decreasing responsiveness to *Trichoderma*‐induced systemic resistance (Jaiswal *et al*., 2020). Iterative rounds of breeding for properties of commercial value progressively uncouples plant responses to health beneficial soil microbes. This reduction in inherent robustness of the crop plant is compounded by the increased susceptibility of monocrops to disease and pest attack, resulting from the unfavourable population dynamics produced by high densities of cultivation and lack of diversity. This in turn entrains a high dependence of such crops on health‐protecting agrochemical pesticides. This unholy coupling of plant breeding of monocrops and increasing agrochemical use has grave consequences for soil health.
  What is clearly evident is that soil (but also biosphere) health is coupled to biota diversity, and that for a very long time, human activities have resulted in major losses of biodiversity, with many species being lost though extinctions. This must be slowed and ultimately stopped through programmes and incentives that focus on the preservation of biodiversity. In the case of soils, plant diversity plays a significant role in assuring microbial diversity, via the holobiont, and the holobiont plays a significant role in determining the diversity of higher forms of life in and on soils. Plant diversity thus plays a key role in maintaining biodiversity, so special efforts must be made to nurture plant diversity.
  Until recently, the soil has been largely viewed as simply a medium/substratum that provides water and minerals to the crop plant. We now need a completely new vision of global policy, with plant benefits to soil health (e.g. soil inputs – quantitative, qualitative, root morphology and influence on soil structure, stability, etc.) as a major focus, with an integration of relevant ecosystem parameters. This will involve acquiring a comprehensive overview of key biota and ecophysiological parameters involved in determination of soil health, soil health‐relevant flora–fauna–microbe–climate interactions, the development and adoption of land‐use practices that respect lessons learned from such research, and so forth.
  Specifically, we need a coordinated global research programme to
  - investigate the genetics of older versions of crops that are more robust and need lower agrochemical inputs, and to focus future breeding^2^ on re‐acquiring those traits responsible for robustness and soil improvement
  - identify the best plants – food monocrops, crop/fallow cover, plants for restoration of degraded soils, etc. – for the best care/restoration of different soils in different climatic zones
  - identify plant combinations that provide synergies in terms of plant and soil health (also, e.g. for cropping regimes, like intercropping/alley cropping (Wolz *et al*., 2018), etc.)
  - obtain a comprehensive overview of plant diversity, conserve it, and explore and exploit it for soil health‐giving properties
2. *The microbes: a focus on plant–soil beneficial properties*.
  - Healthy plants are healthy holobionts: plant:microbiome partnerships. Microbiomes are in part acquired vertically through seeds (Abdelfattah *et al*., 2021), and in part from indigenous soil microbiota. While the same plants in the same area of soil may have microbiomes sharing commonalities, they are not identical because of stochasticity in the acquisition of ‘founder’ (e.g. Litvac and Bäumler, 2019) and keystone (e.g. Mills *et al*., 1993; Banergee *et al*., 2018; Herren and McMahon, 2018; Gibbons, 2020; Chng *et al*., 2020) members, and the subsequent formation around these of effective functional guilds, a process that almost certainly involves eventual rejection over time of less well ‘fitting’ members and their replacement by others. Thus, the possibility of creating /selecting better microbiomes than those that assemble spontaneously exists, as does the possibility of being able to transfer these, or key members of them able to create desired microbiomes (e.g. via microbiome‐coated seeds), in order to be able to establish or restore such microbiomes.
    A significant effort is therefore needed to
    - identify key microbiome members able to create optimal holobionts of different plants suitable for diverse soil and climatic settings
    - identify and understand key functional interactions and regulatory processes that make the plant:microbiome partnership systems work, and that underly the soil care properties, so that these can be influenced to obtain maximal benefits for the hosting soils.
    - identify, investigate and understand the factors that promote instability, the deterioration of healthy partnerships, and the development of unhealthy partnerships, in order to develop strategies to counteract the development of unhealthy holobionts and soils
    - perform holobiont ‘stress tests’ representative of prevailing stresses to identify the best combinations of plants and microbiomes
    - extend research from model plant holobionts in the laboratory and monocrops in the field (Walters *et al*., 2018), which is the basis of current understanding, to natural flora and forest tree holobionts that are the most effective in creating and maintaining healthy soils, and in restoring degraded and desertified/desertifying soils.
    - Trial such holobionts in the field, analyse the soil care properties of optimized holobionts and quantify soil care ‘values/benefits’ in the context of the natural ecosystems, investigate interactions with other soil inhabitants like other microbes and invertebrates and identify optimal partnerships
  - Bulk soil microbes. Although an important component of microbial life in soils is associated with plant roots, most of it is free‐living, carrying out functions vital to soil health (Fierer, 2017), such as biogeochemical cycling (Crowther *et al*., 2019) and the creation and maintenance of disease‐suppressive soils. Key functions of bulk soil, and of individual components of bulk soil, need to understood in terms of the primary organisms involved, and their roles, dependencies on, and interactions with, other members of the soil biota, the plant organic carbon conveyer belt, the root:rhizosphere complex, and the soil components themselves. The goal of this work must be to obtain a conceptual understanding of key processes and the parameters that affect them, predictive models for soil quality and health deterioration, and actionable guidelines for prevention and treatment of ill health.
    This type of research will undoubtedly yield important new insights into which biotic functions are beneficial for soils, and how they can be best cared for. It will also create a wide range of plant holobionts optimal for improving health of diverse soils (Fuller, 1975) that can be used to promote/restore their health. And it will provide new perceptions of soil–biota interactions that will colour attitudes and guide policies to conserve and restore agricultural and non‐agricultural lands.
*In exploring potential strategies to improve and restore soils, the wide range of known beneficial plant:microbe interactions suggests enormous potential for the design and creation of new and improved partnerships for desired functionalities, especially, but not limited to plant holobionts able to grow on and enrich land that is marginal, either because of its natural stresses (composition, latitude, low rainfall, flooding, volcanic activity, etc.), or man‐made stresses (pollution, desertification, etc.), and restore and stabilize degraded land and save it from desertification and erosive loss*.
  - Soil microbe‐based agricultural biotechnology. As already mentioned, soil microbes have been successfully mined for many diverse applications that include symbiotic and non‐symbiotic N_2_‐fixation, protection of and acquisition of soil minerals by plant roots through mycorrhizal fungi, protection from pathogens by antibiotic producing bacteria, like *Pseudomonas* (van Peer *et al*., 1991; Roca *et al*., 2013) and *Bacillus* (Perez‐Garcia *et al*., 2011; Chen *et al*., 2020b), protection from insect pests by entomopathogenic bacteria and fungi, like *Pseudomonas* (e.g. Ruffner *et al*., 2012), Bt toxin‐producing *Bacillus thuringiensis* (Federici, 2005) and fungi, like *Beauveria bassiana* (Ownley *et al*., 2004), increasing root growth by production of plant hormones, inducing systemic resistance by production of volatile signals, increasing tolerance of stresses (de Zelicourt *et al*., 2013), degradation and immobilization of soil pollutants, restoration of soil functionality after fire damage (Pizarro‐Tobias *et al*., 2015; Schenkel *et al*., 2019; Steindorff *et al*., 2021), etc. Many of these, and other, microbial activities can be exploited in applications designed to replace or reduce the use of agrochemicals. There is undoubtedly much more to discover in the soil microbiome and apply in the general areas of plant and soil health (Maestre *et al*., 2017), and efforts to explore and mine this treasure trove of diversity will certainly be rewarded with success.
3. *The soils: a focus on maintenance of health, restoration/reclamation of degraded soils, and prevention of soil losses*.
  One of the problems that soil health analysis faces is the paucity of unified criteria on quality parameters and their levels, together with highly fragmented information on the characteristics of soils in different regions, their fertility and their biological activity (e.g. see Bünemann *et al*., 2018; Bonfante *et al*., 2020). In Europe, through the LUCAS program, important work is being done to harmonize the data, and represents the largest harmonized open‐access data set for topsoil for the European Union, but the level of resolution needs to be improved and expanded (Orgiazzi *et al*., 2018).
  Pro‐active soil health care requires a healthcare mentality of the stewards of the land, a knowledge‐materials‐incentives‐regulatory framework, and adequate organizational infrastructure for the task in hand. At the core of this should be the classical diagnosis – prophylaxis (protection) – therapy (restoration) healthcare pathway.
  - Diagnosis. A healthy soil has plants, animals and microbes functionally interacting in mostly harmonious, coherent unison to maintain all of the usual ecophysiological cycles and activities. Especially given the connectedness of the soil biosphere, and the possibility of wider dissemination of unhealthy developments from individual foci (domino effects; tipping points), it is essential to develop new and sensitive diagnostic technologies and tools for monitoring key functional parameters of soil degradation, and tipping events that accelerate degradation, in particular biological dysbiosis. For this, stakeholders need to specify soil health indicators and their thresholds that define healthy soils. To some extent, soil health/dysbiosis is still a black box, with determinative parameters and underlying mechanisms remaining to be elucidated. The tools and technologies developed to assess soil health therefore need to be informative, i.e. deliver data and knowledge on relevant physico‐chemical and biological parameters, such as microbial diversity and the numbers and activities of keystone microbes of healthy soils (see also Banergee *et al*., 2018; Fierer *et al*., 2021), and thereby contribute to our understanding of what underlies dysbiosis. And tools and instruments need to be inexpensive, simple to use, and suitable for field applications (e.g. Brodie *et al*., 2006; Parro *et al*., 2011; Jacquiod *et al*., 2013).
  - Prophylaxis: maintaining wellbeing.
    - *Agricultural practices and land management*. For the reasons given above, monocrops are problematic for soil biodiversity and hence health. Several crops of monocrops per year amplify the problem, not only because of the biotic effects, but also because the multiple events of mechanized abuse of soils – tillage/destruction of soil structure/drying/exposure of soil to erosive forces and compaction (Troldborg *et al*., 2013) – and because of the multiple applications of agrochemicals. Since monocrops are needed to feed the growing world population, we must counteract their negative impact by (1) reducing the number of crops per season, (2) adopting soil protective practices, like intercropping and alley cropping (Wolz *et al*., 2018), (3) reducing agrochemical use and replacing with soil‐friendlier alternatives, like microbial fertilizers‐pesticides, probiotics and agents that boost plant immune responses to pests (e.g. Thomas *et al*., 2020), (4) reducing mechanized abuse, (5) immediately planting cover plants after harvest, to reduce erosion and provide time and resources for soil diversity to recover between crops, and (6) eliminate stubble burning.
    - *Development of soil protective/regenerative monocrops*. Importantly, we need to develop a range of new monocrop holobionts whose design is not so much for maximal growth and yield but more for improving soil health and fostering belowground microbial diversity to increase the resilience of ecological networks in agricultural land. And we need to find plant partners for such crop varieties that are both soil beneficial and increase crop resilience to disease, and that are useful for intercropping/alley cropping (Wolz *et al*., 2018).
    - *Water supply and moisture‐conserving practices*. A healthy soil has a reasonable water content; aridity reduces microbial diversity and metabolic activity, and decreases resilience to erosion. Efforts must be made to sustainably provide an adequate supply. Equally, it is essential to retain water delivered by irrigation or rainfall, through appropriate ground cover that reduces surface evaporation, and an organic‐rich soil structure that has a high water‐holding capacity.
      Paradoxically, some agricultural soils are overwatered, through sprinklers that operate independently of soil humidity and precipitation events, resulting in both a waste of valuable water and unnecessary washout of soil nutrients. There is an urgent need for inexpensive smart sprinkler systems that provide only the minimum of water, as and when it is needed.
      Water is of course pivotal to the health of arid zones and often subject to competing national interests where a benefit in one country is enjoyed at the cost of prejudiced soil health in others. It is crucial, therefore, to assess and plan the optimal use of water (rivers, lakes and aquifers) at the international level, to promote agriculture, energy generation, job creation, and so forth, in order to achieve optimal global soil conservation and health. This will sometimes involve difficult international negotiations, which also emphasizes the need for UN leadership, and will require integration in other discussions to bring into play meaningful incentives.
    - *Pre/probiotics to maintain healthy soils*. Healthy soil structure and function, and soil stress resilience, are promoted by the regular input of organic carbon and use of organic fertilizers, like plant litter, compost, biochar and a host of solid organic wastes. Increasingly, dedicated crops (green manure), including microbial biomass (Spanoghe *et al*., 2020), are grown specifically for this purpose.
      While maintaining healthy soils will generally involve well tried and tested traditional good land management practices, as our understanding of functional relationships between soil, climate, plants and microbes increases, so will our ability to make more precise, targeted science‐based interventions to prevent deteriorations in soil health. This may include the addition of particular keystone microbes (Banergee *et al*., 2018) playing functionally important roles in healthy soil food webs, microbiome metabolic networks and biogeochemical cycling, either alone or in combination with other products, like biochar.
      The addition of specific microbes is equivalent to administration of probiotics; addition of soil health‐enhancing and soil‐fertilzing materials like green manure, compost and biochar is the equivalent to the administration of prebiotics. And both correspond to the adoption of healthy lifestyle regimes in human health care.
  - Therapy: treating sick soils. Soils that are unhealthy require therapies. And new approaches and materials are urgently needed to develop effective treatment pathways, most of which will involve efforts to create the environmental conditions required to restore and subsequently sustain healthy biological functioning of ‘patient’ soils, particularly their plant holobiont and bulk soil microbial diversity and ecophysiology. In future, it will be crucial to advance to a mechanistic understanding of improved soil functioning, with knowledge of the participating components and their contributions, in order to specifically target individual causes of ill‐health and soil deterioration with tailored remedies (precision medicine). These will include the application of microbially inoculated organic materials, like biochar, as transplantation treatments, addition of keystone species known to be both functionally essential (play key functional roles in healthy soils) and key founder members and effective recruiters of functionally important microbial guilds/microbial guild‐plant partnerships/microbial guild‐based food webs. Depending on the health/degree of degradation of the soil/its fertility, it may be beneficial to transplant soil microbes or plant holobionts from a related but healthy soil growing the same plants. It will undoubtedly be instructive in this endeavour to consider parallels in the field of human medicine, such as the use of microbial transplants to treat inflammatory bowel disease and obesity, where microbiomes play a significant role and are inspiration for prophylactic and therapeutic interventions, and generically beneficial microbes like, *Akkermansia*, are being identified and trialled (de Vos, 2013; Depommier *et al*., 2019; Shetty *et al*., 2019; Korpela *et al*., 2020).
    Treatment will involve restoration of the key elements of healthy soils, i.e. an adequate and reliable supply of water, provision of appropriate plants/trees that will provide ground cover, litter, and a root matrix that provides soil stability and the exudate nutrients that feed microbial life. Microbial life will, in turn, support the plants. The same may be true for soil animals. In some instances, it will be crucial to remove the problem causing ill‐health, such as pollutants.
    - *Pollutant removal*. Pollutants must be removed from polluted soils and an excellent range of remediation technologies exist for this purpose. While all contaminated soils must be targeted, inner city brownfields should receive special attention, and their sealing and use for building should be avoided where possible (see below).
      Specific microbes and specific plant rhizosphere:microbe partnerships, provided with appropriate conditions, can effectively degrade many organic agrochemicals and industrial pollutants (e.g. Timmis *et al*., 1994; Böltner *et al*., 2008). Bioremediation may involve adding nutrients and/or electron acceptors that limit the rate of degradation (biostimulation) or adding specialist microbes, if the catabolic activity itself is absent or limiting (Rojo *et al*., 1987; Erb *et al*., 1997; bioaugmentation). In cases where a pollutant is significantly toxic for much of the biota, a single type of microbe (or consortium containing a particular specialist) able to degrade the pollutant may provide toxicity protection to the entire biota in the immediate vicinity, including macro‐organisms, a phenomenon termed bioprotection (Erb *et al*., 1997). The addition of limiting materials – nutrients, microbes – is analogous to medication. In instances where the pollutant is relatively localized (a point source) and impractical to remediate on site, it may be necessary to physically remove the polluted soil – analogous to surgery – and either treat off‐site or deposit in a hazardous waste disposal facility.
      Some pollutants, such as metal and metalloid compounds, are not degradable, but can be transformed by soil microbes to other states, e.g. valences, which are less toxic or less soluble in water and hence less bioavailable. An example is the conversion of toxic mercuric chloride or organic mercury to essentially insoluble and thus weakly bioavailable and weakly toxic elemental mercury (Horn *et al*., 1994; Wagner‐Döbler *et al*., 2000). In addition, some plants have exceptional ability to take up and store toxic metals, so can also be used to remove pollutants from soils (phytoremediation).
      The ability of microbes and plants to destroy, immobilize or extract pollutants in soil has enormous potential for efforts to restore and reclaim land polluted by historical industrial activities. The performance of such bioremediation catalysts may also be increased by biochar (Gutierrez and Coulon, 2020), a carbon‐rich material obtained by pyrolysis of waste organic matter. Biochar and compost can greatly increase the recovery potential of sick soils (Houses of Parliament, 2010; Chew *et al*., 2020).
    - *Holobiont therapy*. It is becoming increasingly clear from studies of the human microbiome that perturbations of microbiome:host (the holobiont) interactions not only are associated with a multitude of medical conditions previously thought to be microbe‐independent, but that a diverse ‘healthy’ microbiome is important for general health, i.e. for the holobiont partnership. This is for example reflected in the current research into the use of faecal microbiota transplants as a means of restoring a healthy gut microbiota (de Vos, 2013; Depommier *et al*., 2019; Shetty *et al*., 2019; Korpela *et al*., 2020), especially after interventions involving major perturbations of microbiomes through antimicrobial therapy. In some cases, soil microbiota or soil holobiont transplants may also be effective for improvement of soil health. In any case, the diagnosis‐prevention‐treatment pathway needs to be applied, considering the holobionts in the system.
    - *Disease conducive soils*. Disease‐suppressive soils, in contrast to disease conducive soils, are ‘soils in which the pathogen does not establish or persist, establishes but causes little or no damage, or establishes and causes disease for a while but thereafter the disease is less important, although the pathogen may persist in the soil’. (Baker and Cook, 1974). Two types of disease suppressiveness have been described, both due to microbial activities (e.g. Mendez *et al*., 2011). General suppressiveness results from the competitive environment of a high and active microbial biomass, which limits access of the pathogen to available resources. Specific suppressiveness is due to the action of particular microbes that interfere with one or more specific stages in the life cycle of the pathogen, for example by production of antibiotic compounds (e.g. see Schlatter *et al*., 2017). Importantly, specific suppressiveness can be transferred to and conferred upon disease conducive soils. This implies potential for the development of effective disease‐suppressive microbial cocktail therapies for the treatment of conducive soils (and of soil probiotics to prevent the development of conducive soils).
    - *Surface crust formation and water retention*. Soil crusts are biological assemblages containing varying proportions of cyanobacteria, other microbes, algae, lichens and bryophytes (mosses, etc.) that develop on arid soils. It has been calculated that soil crusts cover some 12% of the Earth’s landmasses, where they account for a significant proportion of primary production and provide key ecosystem services, including carbon sequestration, nutrient cycling and climate regulation (Meier *et al*., 2018). Soil surface photosynthetic members of crusts capture solar energy and use it to convert inorganic CO_2_ to organic compounds, some of which consists of secreted polymers that glue together soil surface minerals, forming a crust that stabilizes the surface and acts against erosion, and nurture other microbes in the crust (e.g. Meier *et al*., 2021). Importantly, crusts reduce water evaporation, which is key to microbial diversity and metabolism, and soil productivity. Creating conditions that favour cyanobacterial growth, and seeding with particularly effective crust‐forming cyanobacteria and cyanobacteria‐containing microbial/plant:microbe consortia, is one potential strategy in the arsenal of means to conserve marginal soils and ultimately create conditions for their restoration.
    - *Desealing: the need for an audit*. Sealed soils are functionally dead, but can be resuscitated if unsealed and provided with appropriate care. It is essential to perform, nationally and internationally, a critical audit of sealed surfaces to determine which are essential and which could be desealed and restored. It is equally essential that any proposal for new sealing be convincingly justified and, if justification is of a temporary nature, be accompanied by a specific obligatory desealing plan.
    - *Global solutions to global problems*. While much effort will be directed towards solving local cases of unhealthy soil problems, it is important to seek global solutions (e.g. de Lorenzo *et al*., 2016; Conde‐Pueyo et al 2020), using the powerful new technologies currently available, such as synthetic biology, as well as those in development.

## Knowledge‐based long‐term soil stewardship: the need to integrate into the tapestry of interconnected planetary issues and involve key stakeholders in duty of care. (‘*We are part of the earth and it is part of us… What befalls the earth befalls all the sons of the earth’*. Chief Seattle, 1852)

1. *Interconnectedness of Grand Challenges*. In order to establish a trajectory of sustainable soil improvement progress, it is crucial to treat the soil crisis and its management as part of a complex of interconnected global challenges that include global warming‐extreme weather events, growth in global population and the increasing demands it makes on natural resources such as food, water, energy, etc., agricultural and industrial activities that pollute, the nature and risks of global trade/supply chains/security and its links with poverty‐wealth‐desire for cheap bargains‐rising profits, and so on. Perhaps the most obvious example of this is the fact that soil erosion will not be seriously and sustainably impacted without serious efforts to counter global warming: forests are important buffers against global warming, extreme weather events that lead to soil erosion, and soil erosion vulnerability, quite apart from constituting major soil‐stabilizing agents in their own right. The excellent work of the Food and Agriculture Organization of the United Nations (FAO) in providing comprehensive up‐to‐date information on global soils (http://www.fao.org/soils‐portal/en/) is central to future duty‐of‐care for soils.
2. *Stakeholders.* All stakeholders need to be actively involved in soil health and conservation. For this to be effective, it is essential that they are informed and educated. Farmers, as key stewards of soil health, need to be educated in soil health, which practices contribute to ill‐health and why, and which ones promote good health, and they need incentives to implement recommendations, in particular, adequate revenues for the produce they generate while fulfilling their duty of care for the land they nurture. Gratifyingly, progress on this front is evident in numerous countries and many farmers are responsible stewards of the land, with a highly developed sense of duty of care and determination to leave their land to the next generation in a better state than it was when they assumed responsibility for it.
  But the general public also need relevant information, in order to be able to effectively exercise their stakeholder rights and responsibilities regarding scrutiny of policies and holding policymakers and their agencies to account. In all cases, the most effective means of information transfer is education in school, through the incorporation of relevant topics into school curricula, in order to create a soil literate society. Efforts to create such literacy in microbiology, including in plant holobionts and soil bioprocesses, have been initiated (Timmis *et al*., 2019, 2020; McGenity *et al*., 2020).
  Another key element in successful restoration of soil health is the knowledge of how new agricultural approaches will integrate within the greater landscape, its ecology and sustainable evolution (see also Gann *et al*., 2019; Moreno‐Mateos *et al*., 2020), as well as how they will impact recreational, socio‐economic and cultural factors, including employment creation (permaculture/regenerative agriculture; see e.g. Rhodes, 2017). All of these can greatly affect the sustainable adoption of the strategies and help to ensure balanced rural‐urban growth. Just as a healthy soil requires cooperation between many biological partners, so reducing soil degradation, and conserving the healthy soils we still have, will require dedicated cooperation between many human stakeholders and, importantly, global engagement and cooperation. That this is possible is demonstrated by current international cooperative efforts to mitigate the global warming crisis.
3. *Linking security of food supply with soil care by expanding domestic food production: democratization of soil care*. The supply of most food for humans depends on one hand on agriculture, which depends on soil, and on the other, on distribution networks and supply chains. Distribution networks and supply chains are global, because economics favours production in countries where it is least expensive and to extend product availabilities over the seasons. As a result, food delivery is subject to varied uncertainties along supply chains, such as poor crop yields due to unfavourable weather, soil degradation resulting from practices to increase yields and profits, poor food quality due to contamination with pathogens, pollutants or deterioration due to inappropriate conditions of handling, storage, shipment or distribution, and shortages due to economic (sales to the highest bidders) or political (sanctions, tariffs) actions.
  With economics at the forefront, food production has been outsourced to varying degrees by many countries, even when domestic capacity exists. The consequence is that there is no perceptional link between food and soil in the minds of most people and, even when the link is apparent, it is someone else’s soil. The abuse of land to extract maximum food crop yields, deforestation to create more agricultural land to exploit for quick profit, and the selection of crops with optimal transport and shelf‐life characteristics, rather than flavour and nutritional content, ultimately results in undesirable trajectories of soil degradation and loss, and questionable food quality and diversity.
*We outsource food production and lose control of food quality, diversity and security, and leave the fate of soil to others who may not be subject to considerations other than quick and maximal profit in the short term*.
  Superimposed on these and other considerations that directly impact on food security, is the issue of acute and chronic poverty in some countries, which are unable to provide their citizens with adequate supplies of food, and poverty of individuals, who are unable to buy food even when it is available.
  - Policymakers. A primordial responsibility of policymakers (national, but also international e.g. EU, regional, local), heads of organizations, businesses and families, is to protect their constituents from harm. A primordial need of all forms of life is food, an inadequate supply of which leads to starvation and harm to health. Thus, it is incumbent on all levels of policymakers to take all possible steps to reduce food insecurity. While self‐sufficient domestic food production will not be possible for many nations, an increase in domestic food production, with the attendant advantages of increased food security, control of soil care, etc., is possible in many. But a greater reliance on local production means, on one hand, creating where possible more national farmland, especially through reclamation of degraded lands, and dealing with the issue of increased food costs, where this applies, and increased seasonality of available produce. But any increased costs must be balanced against improved food security, shorter supply chains which means fresher food, higher diversity, including local specialities, of many items, higher quality, and increased support of local industries.
  - General public. There is a perception among many people in high‐income countries that food is something that comes from a supermarket; the existence of a supply chain and what happens in it, if at all considered, is nebulous, abstract. The link between food and the soil we walk on does not exist in many, and the notion that the soil we see from our window might produce fruit and vegetables tasting better than what is on offer in the supermarket is mostly only alluded to in passing in foodie TV programmes. Thus, the perception of soil in our collective psyche needs to change, not only from ‘dirt’ to a living entity needing nurturing, but also that soil within sight or reach could be the growth medium of diverse vegetables and fruit characterized by outstanding flavour and freshness. In regions of the world experiencing food shortages, soil/land must be perceived as an opportunity to reduce such shortages. In both cases, a reduction in dependency of imported food, and the accompanying increase in food security, can be achieved.

While the production of food for self‐consumption in gardens and allotments is normal in rural settings, it is now a rarity in urban settings where > 50% of the population live. And, while gardening does not have the convenience, pizzazz and hype of jogging/visits to the gym, it is a healthy and varied form of exercise, exposes to fresh air and certainly enriches microbiomes. Importantly, it involves new and rewarding experiences: getting in contact with soil, learning about soil biology‐health‐nurturing, experiencing the wonder of seed germination, plant growth and the satisfaction of harvesting crops and consuming them. And, especially in garden communities, such as allotments, gardening also promotes considerable social contact, fosters a spirit of sharing knowledge, materials and produce, and hence is beneficial, especially for those experiencing anxiety, suffering from mental handicaps and/or learning difficulties. Given the human health‐giving properties of consumption of fresh healthy food, exercise, fresh air, exposure to rich microbiota diversity, the pleasure of discovery, and the stress‐counteracting – for some, even meditative – effects of exercise, immersion in nature, and fellowship with other gardeners, growing your own food must be considered to belong to One Health.

Of course, the engagement of people in efforts to grow food for self‐consumption must be supported by measures to provide citizens’ gardens or vertical gardening possibilities, where it is not available to target groups, and education about the health values of gardening and reducing dependence on global supply chains. While growing food for self‐consumption can be promoted through campaigns targeting adults, the most sustainable mechanism is education in schools, which should, where possible, be provided with allotments/vegetable gardens/vertical gardens. Children should have the experience of growing different traditional varieties of tomatoes/apples/plums/etc. and tasting the differences in flavours, one from another and from commercial varieties purchased from the supermarket. And they should have the experience of growing tomatoes in local garden soil, compost‐enriched soil, and degraded soil from a local brownfield and of comparing plant growth.

Thus, (1) national governments should prioritize national food production and the soil protection and reclamation measures associated with this, and incentivize farmers to produce crops best suited to the local conditions and that nurture soil health, (2) local government should provide land for communal use, as allotments or similar, as well as orchards, for public use, and building directives to enable balcony and/or vertical gardening, (3) national governments should enact policies to introduce into school curricula the topic of the health and security benefits of vegetable gardening, also in the context of the global soil crisis, and (4) local governments should incentivize schools to create gardens/allotments on or near the premises to enable children to grow their own food, learn about the theory and practice of the care of soil and plants, and to experience the difference in fruit and vegetables produced locally versus globally.

## Concluding remarks and Recommendations

Soil is a precious and living human good, an important component of our heritage, and that of our biosphere. Its mineral component was formed over many millions of years, its organic carbon is more recent with continuous losses and gains over time, but some was also added over millennia, and its biological component is recent – some was born today. The soil adhering to the carrot we pull out of the vegetable plot is living heritage representing continuity with the distant past, long before we evolved into *Homo sapiens*.

How we treat – mostly abuse – soil is determined by national policies, but the soil crisis is a global problem that does not respect national boundaries (most of which in any case are neither natural nor have a rational raison d’être). Slash and burn policies in one country produce fog and haze pollution over others; coal burning in one country precipitates acid rain and soil deterioration in others. As stated by Leopold, *We abuse land because we regard it as a commodity belonging to us:* that is: as our national/local/personal property, to exploit as we see fit (mostly for short‐term material gain). But we must in future see and treat soil as natural capital (because it ‘provides services of fundamental importance for human wellbeing’: Bardgett and Van Wensem, 2021), a common good, belonging to all, including other animals and the plant world, a common good that needs an effective healthcare system that protects and treats, and effective economic policies, legislative frameworks and adequate education that enable us to provide effective stewardship of our soil heritage (https://enrd.ec.europa.eu/enrd‐static/fms/pdf/260BDE6D‐0066‐3464‐FD34‐E3BB6AD3BB51.pdf; http://www.fao.org/3/ac694e/AC694E06.htm; see also: Ostrom, 2009; https://www.nobelprize.org/uploads/2018/06/ostrom_lecture.pdf, Arrow *et al*., 2012; Bartley, 2021).

However, whereas soil may readily be accepted as a common good, a universal heritage, soil is synonymous with land which, unlike other common goods such as air, water and fisheries outside of territorial waters, is generally in private ownership. But: just because soil may be on land in private ownership, this does not mean that it may be treated inappropriately, any more than people in a village owned by a landowner may be treated inappropriately. Soil stewardship must be subject to higher authority, scrutiny and oversight, and regulation. Moreover, there is an intermediary stage of ‘ownership’: ‘common land’ which, although owned, is accessible to the public or specific subsections of the public for use or recreation/enjoyment (https://www.gov.uk/common‐land‐village‐greens; https://foundationforcommonland.org.uk/a‐guide‐to‐common‐land‐and‐commoning; https://www.commonland.com). Common lands include national, regional and local parks, heritage sites, allotments and grazing lands. Governments, and especially philanthropists, should massively expand common lands, particularly in and near urban areas, in order to create land for diverse uses with a soil health focus, including recreation of various sorts, food production (citizen cultivation of food; citizen fruit orchards), education at all levels – but especially of children (school plots) – with diverse plant covers and roots systems/carbon inputs, including trees, therapy (e.g. animal‐assisted/gardening therapies for those suffering stress and mental challenges; Maber‐Aleksandrowicz *et al*., 2016), plant diversity conservation, pollinator refuges, wildlife refuges, and so forth. The restoration of brownfield sites for this purpose would have the additional benefits of therapy to create healthy soils, inner city location and hence accessibility, and beautification of inner cities. Such common lands could also make a significant contribution to One Health endeavours.

Since soil should be a common good, we will all be stakeholders in the ecosystem services it provides and stewards of its fate. We have argued here that the key problem for society to recognise its obligations is *inter alia* an unholy alliance of the perception of soil as inert 'dirt', a lack of perception of the link between soil and the food supply, a lack of knowldge about ecosystem services provided by soil and its role in numerous Grand Challenges and sustainability issues, and a lack of awareness of the fragility of global soil stocks and their qualities. To remedy this, there is an urgent educational need to help society understand that soil is a dynamic living entity that is our friend and deserving of our care and protection, that its health needs safeguarding by an effective medical system, and that to fulfil our duty of care, we must understand it and what it does, through education, especially in school. We must develop the philosophy of handing over our soil heritage to the next generation in a better state than we received it from the previous one. Effective stewardship of our soil heritage, and the new knowledge, understanding and materials needed for this, international dialogue and policy development, and agencies able to coordinate global action to conserve and restore soils globally, are desperately needed. These include *inter alia*:

1. *Research: soil ‘biomedical’ research programmes*, national and international, to provide new knowledge and understanding of
  - soil health/deterioration indicators and thresholds,
  - the nature and underlying mechanisms of soil beneficial plant:microbiome partnerships,
  - criteria, tools and diagnostic approaches for determining the health status of soils and their plant holobionts,
  - microbial biotechnological options to restore, maintain and improve soil health, the services it provides, and to advance key SDGs, especially 2, 3, 6, 7, 13, 15. (e.g. https://sfamjournals.onlinelibrary.wiley.com/toc/17517915/2017/10/5)
  - conceptual frameworks,
  - living laboratories to develop and test effective and sustainable therapies.
2. *Restoration‐Conservation: soil healthcare systems* – national soil conservation agencies, analogous to public health agencies and healthcare systems, with five components:
  - epidemiology‐monitoring‐forecasting,
  - intervention: diagnosis‐prophylaxis‐therapy,
  - policy development, oversight and regulation,
  - international coordination
  - regulation, authorization and oversight of development and exploitation of products and practices that can influence soil health
3. *International coordination and legislation:*
  - creation of an international (e.g. UN) agency for soil restoration and conservation responsible for developing internationally accepted practices, monitoring progress, and recommending incentives and disincentives for good/bad practices
  - development of an international economic framework to incentivise good soil practices/disincentivise poor ones
  - elaboration of internationally agreed laws to protect the environment, to define ecocrimes/ecocide/environmental crimes, including those that deliberately degrade soil health or pollute, and appropriate sanctions, and creation of the International Environmental Court (https://www.ibanet.org/Article/NewDetail.aspx?ArticleUid=71b817c7‐8026‐48de‐8744‐50d227954e04; Greene, 2019; Solntsev, 2019) to prosecute/adjudicate such laws
4. *Democratization of soil stewardship, education, and activation of stakeholders*.
  - provide education at all levels in soil value, health and loss, in order to create a soil literate society able to adopt, support and insist on policies and measures designed to improve soil health
  - develop policies and practices to increase national, local and family food production based on good soil management principles
  - create a sustainable framework, promote political engagement and local planning that encourages active stakeholder involvement (e.g. by providing garden plots/vertical gardening systems in schools for children/in urban locations for family food production)
  - massively expand common lands, especially in and around urban areas, especially through remediating brownfield sites, and incentivize their productive exploitation and educational application by the urban citizenry

In this Editorial, we hope we have told ‘it like it is’ (Bradshaw *et al*., 2021), without under‐estimating the magnitude, severity and humanitarian and biosphere consequences of the soil crisis. As has been pointed out in the context of other crises, optimism (which encourages failure to recognize seriousness and scale), human behaviour (which favours procrastination over prompt action), the incremental nature of socio‐political processes needed to set solutions in motion, and political reluctance to develop solutions of magnitudes that are adequate to solve the crises, all conspire to allow crises to overwhelm because of remedial action that is too little, too late (see also Bradshaw *et al*., 2021). The soil crisis demands immediate health care in the form of global diagnosis, prophylaxis and therapy. Microbes, microbiomes and microbial biotechnology are ready to play their preordained pivotal roles in soil healthcare interventions.

## Conflict of interest

None declared.

## Footnotes

^1^ Though not directly related to soil losses, it is also important to stress that deforestation also brings (newly formed) farming communities into closer contact with wildlife, thereby increasing the probability of new zoonotic infections, including those caused by SARS viruses (e.g. Field, 2009), which have the possibility of becoming epi/pandemics (Timmis and Brüssow, 2020).

^2^ Breeding is used here in both the classic sense – selection of offspring with desirable traits – and in the molecular genetic sense – genetic engineering/synthetic biology
